# Detection of Mental Fatigue in the General Population: Feasibility Study of Keystroke Dynamics as a Real-world Biomarker

**DOI:** 10.2196/41003

**Published:** 2022-11-21

**Authors:** Alejandro Acien, Aythami Morales, Ruben Vera-Rodriguez, Julian Fierrez, Ijah Mondesire-Crump, Teresa Arroyo-Gallego

**Affiliations:** 1 nQ Medical Inc Cambridge, MA United States; 2 School of Engineering Universidad Autonoma de Madrid Madrid Spain

**Keywords:** fatigue, keystroke, biometrics, digital biomarker, TypeNet, domain adaptation, fatigue detection, typing patterns, circadian cycles, mental fatigue, psychomotor patterns, monitoring, mental health, keystroke dynamics

## Abstract

**Background:**

Mental fatigue is a common and potentially debilitating state that can affect individuals’ health and quality of life. In some cases, its manifestation can precede or mask early signs of other serious mental or physiological conditions. Detecting and assessing mental fatigue can be challenging nowadays as it relies on self-evaluation and rating questionnaires, which are highly influenced by subjective bias. Introducing more objective, quantitative, and sensitive methods to characterize mental fatigue could be critical to improve its management and the understanding of its connection to other clinical conditions.

**Objective:**

This paper aimed to study the feasibility of using keystroke biometrics for mental fatigue detection during natural typing. As typing involves multiple motor and cognitive processes that are affected by mental fatigue, our hypothesis was that the information captured in keystroke dynamics can offer an interesting mean to characterize users’ mental fatigue in a real-world setting.

**Methods:**

We apply domain transformation techniques to adapt and transform TypeNet, a state-of-the-art deep neural network, originally intended for user authentication, to generate a network optimized for the fatigue detection task. All experiments were conducted using 3 keystroke databases that comprise different contexts and data collection protocols.

**Results:**

Our preliminary results showed area under the curve performances ranging between 72.2% and 80% for fatigue versus rested sample classification, which is aligned with previously published models on daily alertness and circadian cycles. This demonstrates the potential of our proposed system to characterize mental fatigue fluctuations via natural typing patterns. Finally, we studied the performance of an active detection approach that leverages the continuous nature of keystroke biometric patterns for the assessment of users’ fatigue in real time.

**Conclusions:**

Our results suggest that the psychomotor patterns that characterize mental fatigue manifest during natural typing, which can be quantified via automated analysis of users’ daily interaction with their device. These findings represent a step towards the development of a more objective, accessible, and transparent solution to monitor mental fatigue in a real-world environment.

## Introduction

### Background

Mental fatigue is a state of brain exhaustion caused by long periods of cognitive activity, lack of sleep, or stress. According to Tanaka et al [1], mental fatigue may lead to overactivation of the visual cortex in the occipital lobe, which has been linked to cognitive impairment and low psychomotor performance. Patients experiencing this condition usually report, among other symptoms, a reduction of their concentration capacity, headaches, dizziness, and slowed reflexes and responses [2]. From a clinical point of view, these psychomotor impairments induced by mental fatigue could be a sign of other emerging diseases, including neurodegenerative or cardiovascular conditions [3,4]. As an example, patients with Parkinson disease have been reported to show higher level of physical and mental fatigue in early stages of the disease than healthy participants [5]. Fatigue has been reported to be one of the major causes of disability for up to half of the patients with Parkinson disease [6], limiting their ability to participate in daily routines or social activities [7,8].

Although multiple tools exist for the assessment of fatigue, there is no clinical standard that enables an objective and complete evaluation of people’s state in this domain. The most accepted method is the Fatigue Assessment Scale, a patient-reported outcome composed of 10 items that evaluate physical and physiological aspects of fatigue [9]. The subjective and episodic nature of these tools makes it difficult to detect and evaluate fatigue in daily practice and in the context of clinical trials. There is a clinical and research need to develop more accessible, accurate, and specific biomarkers to monitor fatigue and its clinical causes [10,11].

Keystroke dynamics is a biometric trait commonly used to authenticate users based on their typing patterns [12,13]. The speed of pressing and releasing keys [14] or the pressure exerted when pressing a key [15] are some of the typing features used by keystroke biometric algorithms for user authentication. Finger kinematics during typing are fine motor skills ruled by the neuromotor cortex and have also been presented as a powerful biomarker in the diagnosis and monitoring of different neurodegenerative diseases, including Parkinson disease [16-18], multiple sclerosis [19], and Alzheimer disease [20]. A recent meta-analysis carried out by Alfalahi et al [21] demonstrates the promising performance of keystroke dynamics–based models for the diagnosis of fine motor impairment in Parkinson disease and mild cognitive impairment diseases. However, the authors show caution in the transition from a controlled assessment in the clinic to the unsupervised remote diagnosis and monitoring, owing to the sparsity and unpredictable nature of typing activity in the real-world context. Continuous keystroke data are easy to gather via commodity hardware (eg, phones and laptops) without requiring the use of proprietary devices. Furthermore, remote data collection can avoid intrusive visits to the clinic, which enhances the patient’s quality of life. Other prior state-of-the-art works have studied how mental fatigue affects typing activity. As an example, Ulinskas et al [22] conducted a study with 53 participants typing a fixed password. They achieved up to 91% of accuracy detecting a state of increasing fatigue between 2 consecutive keystroke sessions by using k-nearest neighbors (k-NNs) classifiers and statistical keystroke features. In contrast, in the study by Slooten et al [23], the authors study which keystroke features are influenced by mental fatigue. They suggest that the addition of keystroke dynamics features to sleep-related markers does not improve mental fatigue detection. However, they point to the subjectivity of the questionnaires used to label the fatigue keystroke data as a limitation to their study.

### Objectives

In this paper, we study the applicability of keystroke dynamics as a potential biomarker of mental fatigue, going a step forward in the state-of-the-art characterization of this psychomotor condition by proposing a new active fatigue detection (AFD) framework based on deep neuronal networks (DNNs). To develop this, we will use TypeNet [24], a state-of-the-art DNN originally designed to model identity via typing patterns at large scale (approximately 100,000 users). The main idea behind this work is to leverage the keystroke dynamics patterns learnt by TypeNet for user recognition and to reoptimize this network for the fatigue detection task.

**Figure 1:**
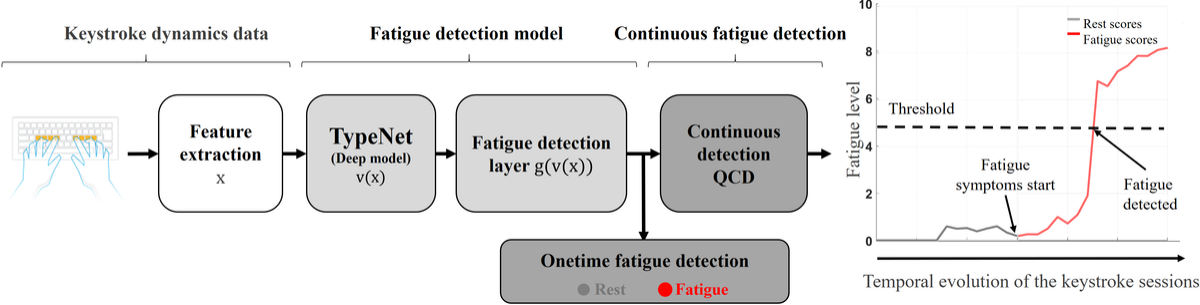
Block diagram of the entire system proposed. The fatigue detection layer adapts the capacity of TypeNet to model user behavior through keystroke patterns for the fatigue detection task. This information is taken by the active detection algorithm to detect changes in users’ fatigue level over consecutive keystroke sessions. QCD: quick change detection.

A schema of the proposed system is shown in Figure 1. The system is composed of 3 main elements: the input layer, the fatigue detection model, and the postprocessing module for active detection. The input layer ingests keystroke session data and generates a predefined feature vector that is then fed to the fatigue detection model. The fatigue detection model is created by connecting the output of the TypeNet network to a fatigue detection layer, which optimizes the original authentication model for fatigue identification. Finally, the postprocessing module for active detection ingests the temporal sequences of fatigue detection scores to produce users’ calibrated fatigue level on the basis of their baseline or previous fatigue states. This block enables real-time monitoring of on-off fatigue fluctuations over consecutive keystroke sessions. In this work, we evaluate the proposed system in a controlled data context to test the performance of the fatigue detection model to discriminate between labeled rest and mental fatigue sessions. In addition to this, we present a real-world application of the system applied to natural typing data to evaluate its suitability to identify daily fatigue cycles in a healthy population.

The main contributions of this work are 4-fold: (1) we develop a deep neural network able to identify mental fatigue symptoms through keystroke patterns, (2) we analyze the ability of the proposed model to detect small variations in fatigue levels between different keystroke sessions, (3) we propose an AFD algorithm that continuously monitors users’ keystroke session sequences to detect longitudinal variations in their fatigue state, and (4) we evaluate the applicability of the proposed system to detect fatigue trends in real-world user data.

## Methods

### Keystroke Data Sets

In this section, we analyze in more detail the 3 keystroke databases (summarized in Table 1) used in this work to train and evaluate our proposed system.

- First, the Aalto database [25] was used to train the TypeNet model that we used as keystroke embedding feature extractor in our fatigue detection model. This database is composed of 168,000 participants with 15 keystroke sessions per participant. The database was acquired using a web-based questionnaire under an uncontrolled environment where each user used their own physical keyboard. All users were initially informed of the acquisition of their press (key down) and release (key up) event timings during the completion of the questionnaire. The questionnaire required users (1) to memorize an English sentence randomly chosen from a pool of 1525 sentences of the Enron mobile email and Gigaword Newswire corpus (these sentences contained a minimum of 3 words and a maximum of 70 characters) and (2) to type the memorized sentence as quickly and accurately as they could. All participants in the database completed 15 sessions (ie, one sentence for each session) on either a desktop or a laptop physical keyboard. The authors of the database reported demographic statistics of the users: 72% of the participants took a typing course, 218 countries were involved, and 85% of them had English as native language. The richness of the Aalto database resides not only in the huge amount of participants acquired but also in the diversity of ethnicities, countries, and different typing skill levels of the participants enrolled allowing TypeNet to authenticate users through keystroke dynamics at internet scale with a high performance [26].
- Second, the neuroQWERTY Sleep Inertia (nQSI) database [27] was designed to detect psychomotor impairment by waking up the participants during the night, thus inducing a sleep inertia status (a mental fatigue condition produced by lack of sleep). The database comprises 14 healthy participants with 4 keystroke sessions per participant of 15-minute duration collected in mechanical keyboards. Two of the keystroke sessions were captured during the day, whenever the participant felt well rested, labeling them as rest state (no fatigue). The other 2 keystroke sessions labeled as the fatigue ones were captured at midnight, when the participants woke up during the phase III and IV of the sleep cycle [28] to capture the keystroke sessions, thereby inducing the sleep inertia state. The acquisition process was monitored by the owners of the database to ensure the quality of the keystroke data captured in both rest and fatigue states (supervised scenario). We used this database to train and test our proposed system for the mental fatigue detection task through keystroke dynamics.
- Finally, the neuroQWERTY Crowdsource (nQCS) database [29] is composed of >800 participants from a healthy control group and group of patients with self-reported neurodegenerative diseases or other conditions (eg, Parkinson disease, Alzheimer disease, multiple sclerosis, or rheumatoid arthritis) typing on mechanical keyboards during a time span of 9 months. An enormous challenge for exploiting this data set is that the keystroke data captured were acquired passively, in a total transparent way for the participant, without any type of supervision or labeled data. In the context of this work, this database was used to study whether our proposed system was able to detect trends in the fatigue levels during the daily typing habits of the healthy participant subset (a total of 251 healthy participants).

**Table 1:** List of keystroke data sets used in this study.

Database	Subjects, n	Sessions, n	Session size	Supervised	Context
Aalto [25]	168,000	15	Approximately 70 keys	No	Development of TypeNet for general user typing model
neuroQWERTY Sleep Inertia [27]	14	4	15 minutes	Yes	Development and evaluation of the fatigue detection system
neuroQWERTY Crowdsource [29]	251	Approximately 1000	Approximately 3 minutes	No	Evaluation of fatigue detection in a real-world environment

### Ethics Approval

Participants in the nQSI study provided informed consent before experiments, and experimental procedures were approved by the Committee On the Use of Humans as Experimental Subjects at the Massachusetts Institute of Technology (protocol number 1311).

Participants in the nQCS study provided informed consent before experiments, and experimental procedures were approved by the Committee On the Use of Humans as Experimental Subjects at the Massachusetts Institute of Technology (protocol number 1504007090).

### Data Preprocessing and Feature Extraction

The raw data captured in all 3 keystroke databases are time series of 3 dimensions: press times, release times, and the keycode of each key. Owing to privacy concerns, the keycode was discarded, and the keystroke features computed for each keystroke session were based only on the press and release key time events. These timestamps were in coordinated universal time format but with different time resolution depending on the acquisition protocol and device used in each keystroke database. To normalize the keystroke data of the 3 databases, all timestamps were converted to seconds while ensuring that all keystroke features computed later are close to 1. This normalization step is necessary to avoid saturation of the neurons in the recurrent layers of our system.

The keystroke features vector is extracted at key level and is composed by (1) hold times (ie, the elapsed time between press and release a key), (2) flight times (ie, the elapsed time between 2 consecutive press events), (3) interkey latency (ie, the elapsed time between release a key and press the next key), and (4) interrelease latency (ie, the elapsed time between 2 consecutive release events). According to this, the keystroke feature vector x used as input of our model has a dimension of 150 × 4 (150 keystrokes by 4 features). If the keystroke sequence is lower than 150 keys, we compute zero padding to fill with zeros up to reach such length; otherwise, we truncate the keystroke sequence taking the first 150 keys.

The reason why we chose these keystroke features is because we wanted to ensure to keep the same feature set as the one used to evaluate the TypeNet DNN model in previous works [24,26,30] with the Aalto database. Remember that the TypeNet model is part of our fatigue detection system that we adapt for the fatigue detection task with transfer learning techniques, and therefore, the keystroke features set used to feed the TypeNet model (ie, the input of our fatigue detection model) must be the same.

### System Design

**Figure 2:**
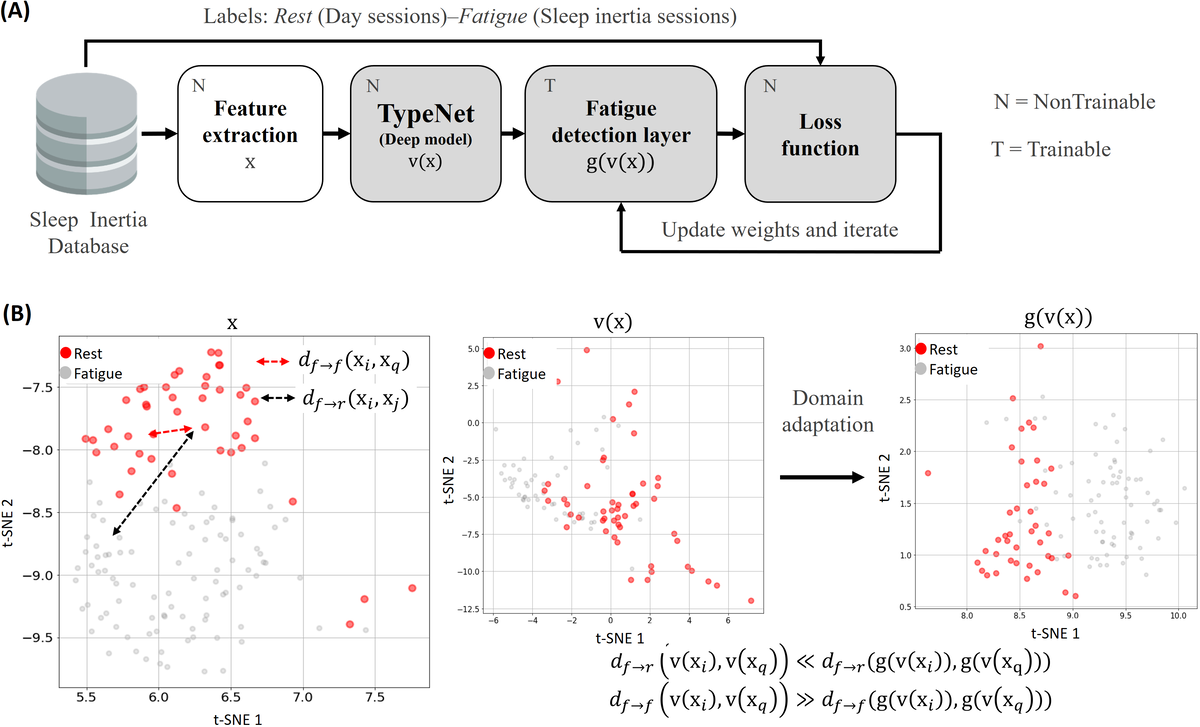
Overview of the fatigue detection model design. (A) The fatigue detection model is trained with the labeled keystroke data from the neuroQWERTY Sleep Inertia database. At the output, the model separates the fatigue embedding vectors g(v(x)) that correspond to each of the 2 user’s states under study (ie, fatigue or rest) while favoring proximity between the embedding vectors that belong to the same class. (B) An example of the transformation from the embedding vectors generated by TypeNet v(x) at the embedding output of the proposed model g(v(x)). The sample output shown in this figure applies t-distributed stochastic neighbor embedding (t-SNE) to generate a 2D projection of the 1×128 output.

**Figure 3:**
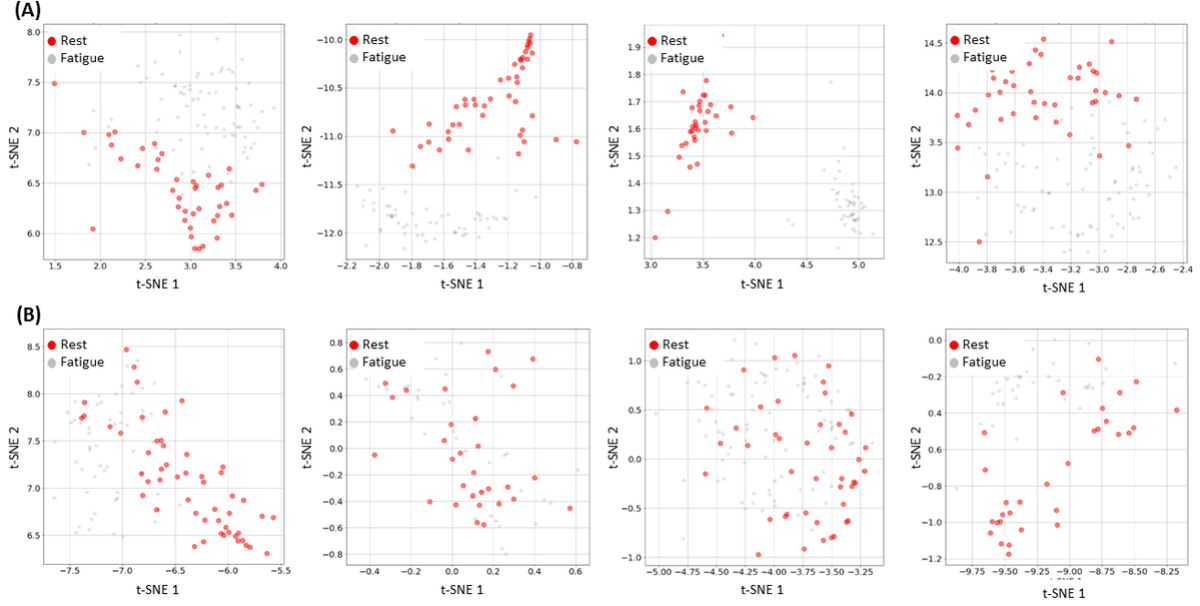
Intrauser variation of the embedding fatigue vectors g(v(x)). We observe how the fatigue detection model presents varying performance depending on the user. Row (A) shows examples of fatigue embedding vectors for those participants where we observe a good separation between fatigue and rest embedding vectors, whereas for participants in the row (B), the separation is not as clear. This user-dependent performance could be a result of the varying levels of intrauser fluctuations observed during natural typing [33]. t-SNE: t-distributed stochastic neighbor embedding.

The fatigue detection model is trained and tested with the labeled keystroke data from the nQSI database. As depicted in Figure 2A, the input of the TypeNet network is a keystroke feature vector x extracted from the raw keystroke data in the nQSI database. The output of TypeNet is a 1 × 128–dimensional embedding feature vector v(x) that authenticates users by applying a distance metric learning (DML) method [31]. TypeNet was originally trained to model the typing patterns of 100,000 users. The training process of TypeNet was aimed to generate a 128-dimensional feature space where keystroke events generated by the same user tend to cluster in a closer region of the feature space, whereas events from different users are projected in different areas of the same feature space. In this work, we use the nQSI data set to adapt the transformed authentication feature space to the fatigue detection task. We apply domain adaptation techniques [32] based on the addition of a fatigue detection layer that is trained to transform the authentication-based feature vectors, v(x), into fatigue detection feature vectors with the same dimension, g(v(x)), as shown in Figure 2B. The fatigue detection layer is optimized using a DML approach and a leave-one-out (LOO) cross-validation protocol. Figure 3 presents some examples showing the results of the transformation in the nQSI data set. The hypothesis underlying the method is that the features learned to model the typing patterns x of 100,000 users contain useful information to characterize users’ fatigue patterns. The fatigue detection layer serves as a nonlinear transformation g(.) to reveal such patterns in the learned space v(x). The fatigue score is computed at the output of the fatigue detection model as the Euclidean distance between pairs of fatigue detection feature vectors (equation 2).

### TypeNet Architecture and Domain Adaptation

The TypeNet architecture proposed in the study by Acien et al [26] is composed of 2 long short-term memory layers of 128 neurons. Long short-term memory layers are a special type of recurrent neural network layers specifically designed to be sensitive to temporal changes in the input sequences, which we think could be well suited to detect relevant changes in the typing behavior of the participant when they are fatigued. In addition, each recurrent layer has a recurrent dropout of 0.2 and a dropout layer of 0.5 between them to avoid overfitting during training. The input of the TypeNet architecture has a masking layer to avoid the computation of error gradients for those zeros (ie, zeros generated when zero padding is needed for keystroke sequences lower than 150 keys) and do not contribute to the loss function during training (more details of TypeNet architecture and evaluation are provided in the study by Acien et al [26]). Finally, the output of the TypeNet architecture is an embedding feature vector v(x) of size 128 × 1.

In this work, we transform this embedding feature vector v(x) (originally used for keystroke user authentication at large scale) into a new embedding vector g(v(x)) of the same size that is better suited for the fatigue detection task. To do this, we use domain adaptation techniques [32], in which the model learns a new task (ie, the keystroke fatigue detection task) via knowledge transfer from a previously learnt task (ie, keystroke user authentication). In Figure 1, an overview of the entire transfer learning process is depicted. The output of the TypeNet model is connected to the fatigue detection layer, which is composed of a multilayer perceptron layer of 128 neurons with *relu* activation. During the training process, the keystroke feature vector x extracted from keystroke sessions of the sleep inertia database is used to feed the TypeNet network, which is frozen during the entire training process so the weights of this network are not altered. Then, TypeNet computes the embedding features vector v(x) that are optimized for keystroke user authentication, thanks to the previous training with the Aalto database in Acien et al [26]. Finally, the fatigue detection layer is fed with this embedding feature vector and learns to transform these embedding features into a new feature embedding vector g(v(x)) optimized for the fatigue detection task, thanks to the labeled data of the nQSI sleep inertia database.

This type of domain adaptation process is also referred to as fine tuning, where the part of the TypeNet architecture that has the knowledge of typing patterns from thousands of users of the Aalto database is frozen, and therefore, we only need to train the last layer (the fatigue detection layer) to adapt these typing patterns for the fatigue detection task with the sleep inertia database. The main reason why we use transfer learning with fine-tuning techniques is because to train a DNN model from the scratch for the fatigue detection task, we will need thousands of participants with labeled keystroke data to make the model robust, generalizable, and accurate. This technique allows us to overcome this issue, taking advantage of other DNN models previously trained with thousands of participants for a similar task like TypeNet, and adapt it for the fatigue detection task using only 16 participants of the sleep inertia database. Fine-tuning techniques have been broadly used in state-of-the-art works [34-36], where the databases used are not large enough to train a DNN model from scratch.

Finally, to train the fatigue detection model successfully, we use the triplet loss function. This loss function is well suited for DML approaches where the output of the model to train is an embedding feature vector instead of a single score. A triplet is composed by 3 different samples from 2 different classes: Anchor (A) and Positive (P) are different keystroke sequences from the same class (fatigue or rest), and Negative (N) is a keystroke sequence from the other class. The triplet loss function is defined as follows:



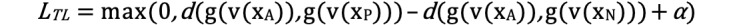



where *α* is a margin between positive and negative pairs and *d* is the Euclidean distance calculated as follow:



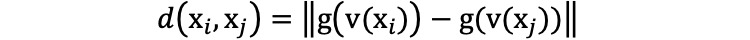



**Figure 4:**
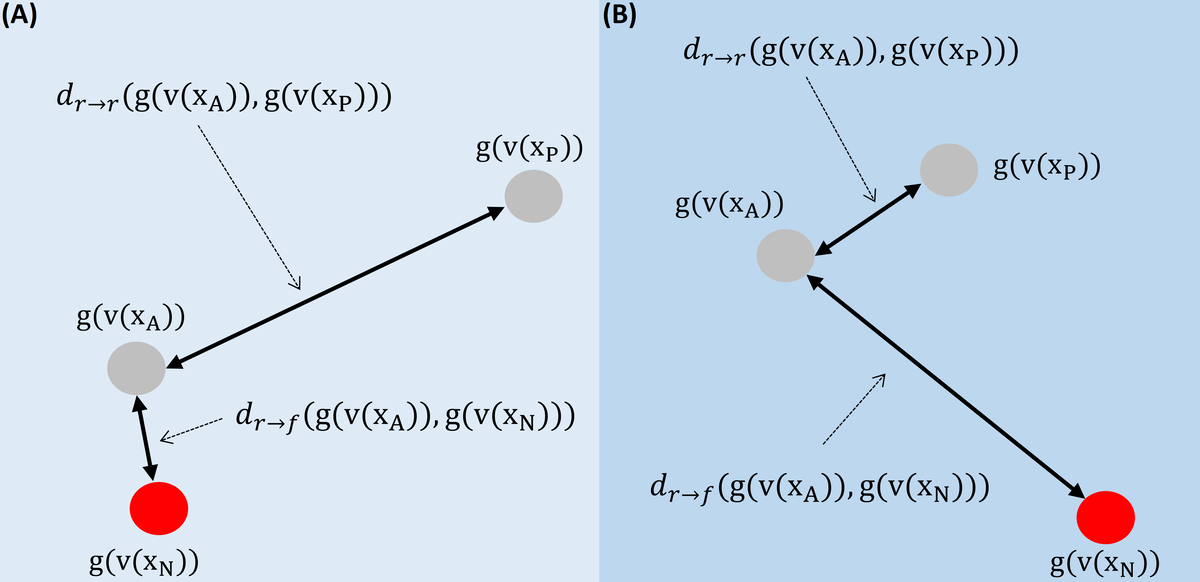
Example of how triplet loss works. 2D representation of the embedding feature fatigue vectors g(v(x)) before (A) and after (B) the triplet loss training. The embedding vectors that belong to the same class (g(v(x_P_)) and g(v(x_A_))) get closer; meanwhile, they get far from the embedding of the opposite class (g(v(x_P_)) and g(v(x_N_))).

This learning process minimizes the distance between embedding vectors from the same class (*d*(g(v(x_A_))*,* g(v(x_P_)))), and maximizes it for embeddings from different classes (*d*(g(v(x_A_))*,* g(v(x_N_)))). Note that all 3 samples x_A_*,* x_P_*,* and x_N_ belong to the same participant to avoid intrauser variations as much as possible. An example of how the triplet loss function works is depicted in Figure 4A, where g(v(x_P_)) and g(v(x_A_)) are 2 feature embedding vectors (ie, the output of the fatigue detection model when fed with x_P_ and x_A_ samples, respectively) that belong to the same class, whereas g(v(x_N_)) belongs to the opposite class. During the training process (Figure 4B), the triplet loss function will make g(v(x_P_)) and g(v(x_A_)) get closer at the same time they get far from g(v(x_N_)). Remember that we only train the fatigue detection layer because the TypeNet network is frozen during training (fine tuning), thereby this entire process is learnt by the fatigue detection layer. The unique purpose of this layer is to separate in the latent space the feature embedding vectors that belong to the rest state from those that belong to the fatigue state. Examples of the final results are shown in Figure 3 by applying dimensional reduction to the embedding feature vectors for 2D visualization. Regarding experimental protocol details, we follow a LOO cross-validation strategy by using all participants but one of the sleep inertia database to train the proposed system and testing with the remaining participant. This means that we have 16 different fatigue detection models (one for each test participant). Regarding training details, the hyperparameters remain the same as those used to train TypeNet in the study by Acien et al [24]: learning rate of 0.005 and Adam optimizer with *β*_1_=0.9, *β*_2_=0.999, and *ε*=10^−8^. The models were trained for 30 epochs with 100 batches per epoch and 64 triplets in each batch.

### Quick Change Detection Algorithm

**Figure 5:**
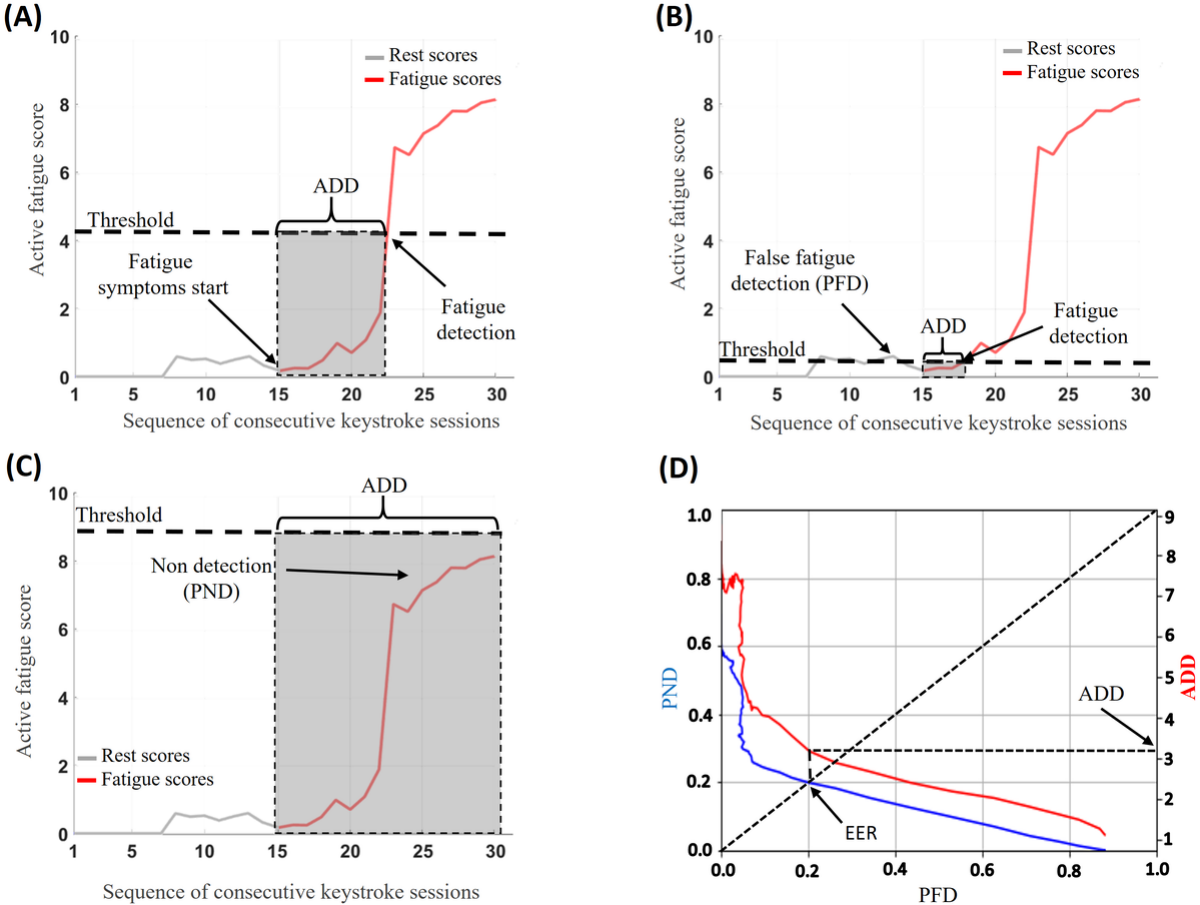
Active fatigue detection (AFD) curves. (A), (B), and (C) are 3 different use cases of the AFD algorithm where the threshold chosen affects the performance. (D) It shows the probability of false detection (PFD) versus the probability of nondetection (PND) and PFD versus average detection delay (ADD) curves as a result of moving the threshold; the value chosen for the threshold is the point where both PFD and PND values are equal, called equal error rate (EER).

The AFD algorithm is based on the quick change detection algorithm proposed in the study by Perera et al [37] for intrusion detection based on mobile behavior biometrics. In this work, the algorithm is redesigned for the AFD task. The algorithm is based on calculating a new score from the cumulative sum of previous events (keystroke sessions). If the participant is in a rested state (gray lines in Figure 5), the cumulative sum will be almost 0. At the moment the mental state of the participant changes into fatigue during typing, this score will tend to increase until reaching a certain threshold, in which we detect the fatigue symptoms. This module can be interpreted as a postprocessing step connected at the output of the fatigue detection model to increase the reliability of the system and to account for the relevance of participants’ preceding states when computing their current fatigue score.

**Figure 6:**
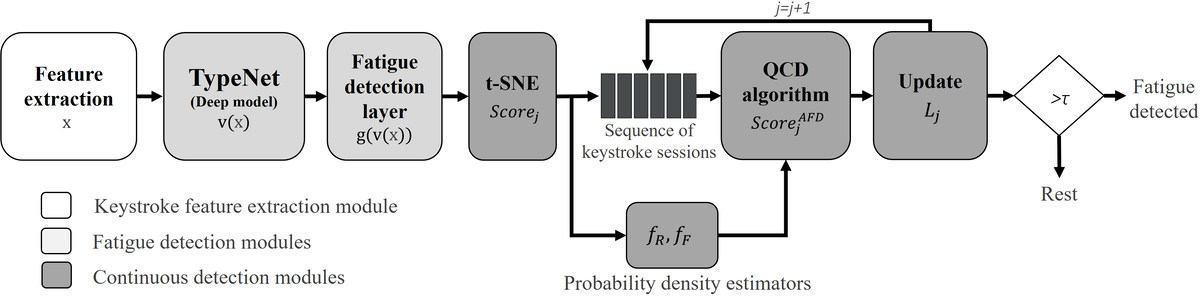
The entire pipeline of the active detection algorithm. The score_j_ is computed by performing t-distributed stochastic neighbor embedding (t-SNE) for dimensional reduction to the embedding fatigue vector g(v(x)). Then, score^AFD^ is obtained by comparing score_j_ with the distributions obtained from the neuroQWERTY Sleep Inertia (nQSI) database. Finally, a threshold τ is used to detect the Fatigue states. QCD: quick change detection.

To evaluate the AFD algorithm, we will use the precomputed fatigue detection scores resulting from the LOO framework that optimized the fatigue detection model. This ensures that the condition of independence between training and testing sets is carried over in this new experiment. In this context, for a given participant, the cumulative sum is calculated as follows:



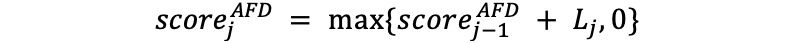



where *j* means the actual keystroke session and 
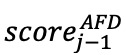
 is the previous cumulative score. *L_j_* is the contribution of the actual event calculated as the log-likelihood ratio between score distributions:



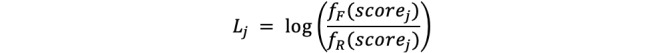



where *score_j_* is the fatigue score of the participant’s current event, and *f_R_, f_F_* are, respectively, the probability density estimators of the participant’s remaining rest and fatigue scores. Note that the output of the fatigue detection model is an embedding feature vector g(v(x)) of size 1×128, so we compute t-distributed stochastic neighbor embedding for dimensional reduction to one dimension (ie, we reduce the size of the embedding vector to one) to obtain a single fatigue score *score_j_*. According to equation 4, the log-likelihood ratio *L_j_* will be negative if *score_j_* belongs to rested keystroke session and positive in the opposite case, and therefore, multiple consecutive keystroke sessions of the fatigued participant will increase the cumulative sum *score_j_^AFD^*. Figure 6 depicts an example of the entire AFD algorithm pipeline for a single participant. The fatigue detection model computes the embedding feature vector g(v(x)) when fed with a keystroke session. Then, we compute t-distributed stochastic neighbor embedding for dimensional reduction to obtain the *score_j._* Finally, we upgrade *score_j_^AFD^* by computing the *L_j_* with the new score according to equation 3, which will increase up to reach the fatigue detection threshold in case the participant is fatigued.

## Results

### Onetime Fatigue Detection Approach

**Figure 7:**
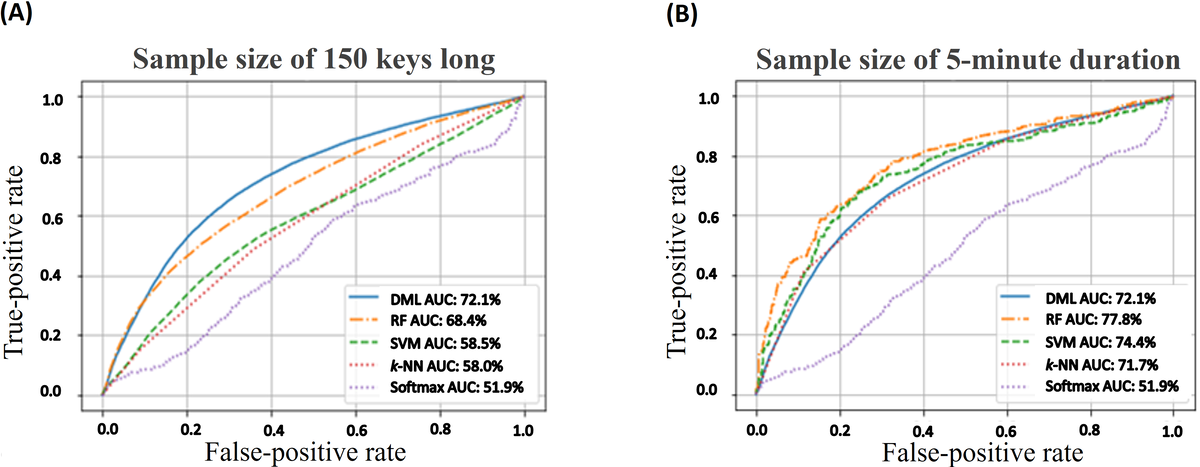
Receiver operating characteristic (ROC) analysis for fatigue detection. Area under the curve (AUC) scores computed with keystroke sample pairs of length 150 keys (A) and 5-minute duration (B). The ROC curves were calculated independently for each participant, and the ROCs showed are the average of all of them. DML: distance metric learning; k-NN: k-nearest neighbor; RF: random forest; SVM: support vector machine.

To evaluate the performance of the fatigue detection model, we use the nQSI data set to generate a pool of intrauser keystroke sample pairs. We contemplate a binary classification framework based on 2 scenarios: (1) no change—when the 2 samples belong to the same class (fatigue→fatigue or rest→rest) and (2) change—when the 2 samples belong 2 different classes (fatigue→rest or rest→fatigue). In Figure 2, we can observe the distances for 2 examples: *d _f_*_→_ *_f_* (x*_i_,* x*_j_*) is the distance between 2 fatigue samples (no change, distance between 2 red dots) and *d _f_*_→_*_r_*(x*_i_,* x*_q_*) is the distance between the fatigue and rest sample (change, distance between a red and a gray dot). The distance between samples is directly compared with a predefined threshold. A fatigue score superior to the threshold reveals a change in the keystroke patterns, whereas a value below the threshold implies no change. We compare the performance of the fatigue detection model based on DML with different statistical classification algorithms trained with the feature vectors x: random forest (RF), support vector machine (SVM) with Gaussian Kernel, and k-NN. In addition, we also compare with the proposed fatigue detection model but replacing the DML approach by a softmax activation layer trained as a binary classification model using binary cross entropy loss. This provides a reference deep learning model used as baseline to compare with our DML approach. Figure 7 presents the receiver operating characteristic analysis comparison in 2 different setups. In the first one, we limit the input size to 150 keystrokes per sample. This input format was defined in accordance to the design of the pretrained TypeNet architecture. In this scenario, the best performance is achieved by the proposed fatigue detection model that achieves an area under the curve (AUC) of 72.1%, followed by the RF classifier with AUC of 68.4%. The worst performance is observed in the softmax-based variation of the proposed fatigue detection model.

In the second set-up, we increase the input size to 5-minute long keystroke sessions (ie, an average of approximately 1100 keys per sample) for the RF, SVM, and k-NN classifiers, while keeping the original 150-keystroke long inputs for the proposed fatigue detection methods and its softmax variation (owing to the limitation of 150 keys as the input size of the TypeNet model). In this case, the DML approach is slightly outperformed by the RF and SVM classifiers that present AUCs of 77.8% and 74.4%, respectively, in exchange of larger input data. Finally, we summarize the performance metrics for the 2 setups proposed in Table 2. We can observe that our DML approach achieved the highest *F*_1_-score, a measure of the test accuracy, in both scenarios. Sensitivity and specificity values are estimated using the closest-to-(0,1) corner in the receiver operating characteristic plane to define the cutoff point. Performance metrics are computed by pooling the cross-validated scores into a single set of predictions used to generate an overall metric estimate for the whole system.

**Table 2:** Performance metrics of the onetime fatigue detection approach.

Set up	System	AUC^a^ (%)	*P* value	Specificity (%)	Sensitivity (%)	Precision (%)	*F*_1_-score (%)
150 keys	Fatigue (DML^b^)	72.1	<.001	73	69	67	72.2
150 keys	Random forest	68.4	<.001	68	63	64.6	70.3
150 keys	Support vector machine	58.5	<.001	58	58	57.9	65.2
150 keys	k-nearest neighbor	58	<.001	77	51	64.6	70.3
150 keys	Fatigue (Softmax)	51.9	<.001	50	52	48	49.1
5 minutes	Fatigue (DML)	72.1	<.001	73	69	67	72.2
5 minutes	Random forest	77.8	<.001	70	76	66.3	71
5 minutes	Support vector machine	74.4	<.001	70	73	65.9	70.7
5 minutes	k-nearest neighbor	71.7	<.001	76	65	64.7	67.6
5 minutes	Fatigue (Softmax)	51.9	<.001	50	52	48	49.1

^a^AUC: area under the curve.

^b^DML: distance metric learning.

### Continuous Fatigue Detection Approach

In this experiment, we consider the quick change detection algorithm [37] that dynamically updates a confidence fatigue score by calculating a cumulative sum from previously measured fatigue states. The purpose of this algorithm is to adapt the fatigue detection method to the needs posed by real-time evaluation of fatigue in a real-world environment.

In Figure 5A, we show an example of the application of this algorithm at the output of the fatigue detection model. The example uses a simulated sequence of keystroke sessions generated by concatenating 15 rest and 15 fatigue keystroke samples from a user in the nQSI database. As the simulated sequence starts in a rest state, the initial fatigue scores are lower and close to 0 during the first 15 evaluation intervals (ie, the 15 user keystroke sessions labeled as rest in the nQSI database). As the simulated sequence starts introducing fatigue samples (from the remaining 15 user keystroke sessions labeled as fatigue), the AFD score tends to increase until it reaches a certain threshold that would indicate there has been a fatigue state change. The number of keystroke sessions elapsed since the models start getting fatigue samples until the AFD algorithm reaches the fatigue threshold is called average detection delay (ADD). This parameter measures the number of keystroke sessions required to detect fatigue since the symptoms start.

The configuration of the threshold in the AFD score is crucial for the performance of the algorithm. As shown in Figure 5B, as we lower the threshold, we reduce the ADD from 7 (Figure 5A) to 3 keystroke sessions, in exchange of a higher risk of false positives. This value is called probability of false detection (PFD) and measures the probability of false fatigue detection (similar to the false match rate). In contrast, increasing the threshold controls the PFD at the cost of increasing the ADD as well as the probability of nondetection (PND). PND measures the probability of the active fatigue score never reaching the threshold over a sequence of keystroke sessions in a fatigued interval (Figure 5C).

According to this, there is always a trade-off between the PND and PFD values as we move the threshold. Figure 5D shows the PND (left y-axis) versus PFD and ADD (right y-axis) versus PFD. To optimize both specificity and sensitivity metrics at the same time, we have the point equal error rate (EER). The EER value is the point where the blue curve (ie, PND vs PFD) crosses the diagonal (the dotted black line) and is equal to 20%. This would be equivalent to an AUC=100−EER=80%. Finally, based on the configuration of the threshold, we can infer the number of fatigue keystroke sessions required, according to our results, to reach the threshold (ie, the ADD value). Once we have calculated the EER that minimizes both PND and PFD values, the red curve (PFD vs ADD) in Figure 5D indicates the number of keystroke sessions required (ADD) for the chosen PFD, which is slightly above 3.

### Independent Evaluation in Real-world Environment

**Figure 8:**
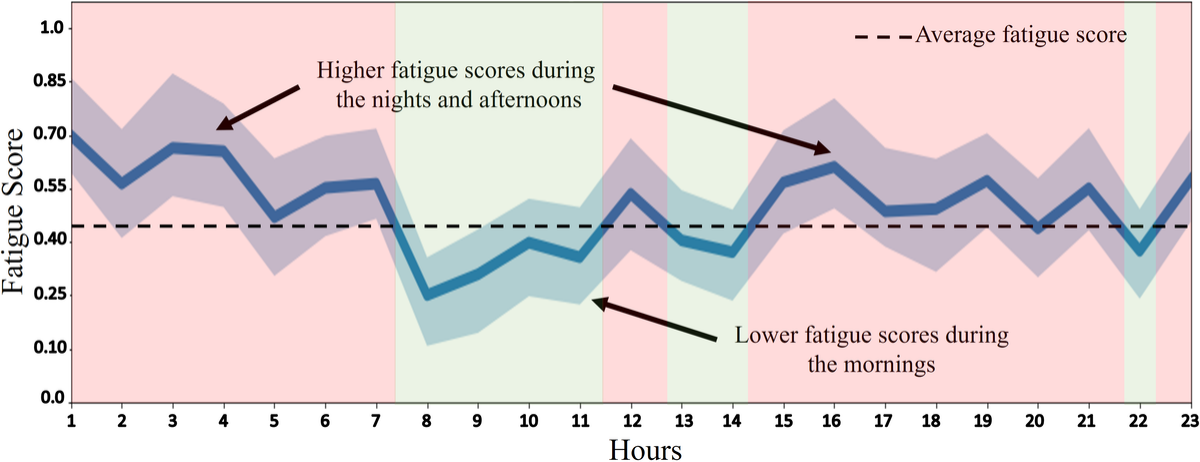
Fatigue score analysis in the neuroQWERTY Crowdsource (nQCS) database. The fatigue scores are calculated, at the user level, between consecutive sessions over their daily typing activity. The graph presents the nQCS population aggregate average and CIs of the resulting fatigue score daily sequences.

As mentioned above, the nQSI database used to evaluate our system was acquired under supervised conditions with labeled keystroke sessions. To evaluate the behavior of the proposed method in the context of its intended use, we applied the resulting model to the nQCS database. As a reminder, this database includes keystroke data from a group of healthy volunteers that was captured during their daily use of the device, without any supervision or prompt to stimulate typing activity. We compute the fatigue scores measured on each pair of consecutive keystroke sessions for each user typing stream. Each user typing stream is composed of multiple keystroke sessions generated over varying observation periods and activity levels. We only take into account the fatigue scores obtained between keystroke sessions with elapsed time of <2 hours within the same day to avoid long pauses between sessions that may introduce artifacts in the resulting fatigue signal. In Figure 8, we present the aggregate trends of the fatigue score levels versus the time of the day. The results suggest lower fatigue levels during the morning and midday hours. Higher fatigue scores are observed during the afternoon hours and overnight. Note that this figure was obtained by averaging the scores from all 251 volunteers in the nQCS database, and therefore, there is an equalization effect caused by different user’s habits.

## Discussion

### Principal Findings

Using domain adaptation techniques, we leveraged an algorithm built for user authentication to detect signs of fatigue via natural typing. The resulting classifier was then adapted for real-time fatigue monitoring by appending an active detection algorithm that compares successive user states. This allows for background evaluation of users’ fatigue state in an objective and real-world environment. The proposed classifier was able to differentiate intrapatient fatigue versus rested states with an AUC of 72.1% in onetime detection set-up. When simulating a continuous detection set-up by concatenating consecutive keystroke sessions, the proposed model is able to detect early fatigue symptoms after 4 keystroke sessions with an AUC of 80%. A preliminary application of the fatigue classifier combined with active detection showcased its applicability to real-world data in a crowdsource data set. Given that this method relies on data collected passively from a user’s daily interactions with their computer, the proposed pipeline operates unobtrusively with low burden and allows for a background, objective evaluation of a user’s fatigue state in the real-world environment.

Relying on machine learning techniques, we were able to liaise a large data set created to study typing behaviors in the general population with the information gathered in a limited size data set built specifically to characterize fatigue through the analysis of keystroke dynamics. This approach allowed us to apply a deep learning architecture in the absence of a high-dimensional data set specifically characterized for the phenomenon under study, quantification of daily fatigue levels in users’ keystroke patterns. Our work exhibits the potential of domain adaptation techniques to minimize the complexity of gathering large and curated data repositories required to train deep learning models by taking advantage of open-source unsupervised data sets in combination with much smaller supervised data sets. In our case, the Aalto database supplies the high volume of data required to build a network optimized for user authentication that is then fine-tuned using the sleep inertia data set to solve the fatigue detection task. Another novel technical contribution of this work is the addition of an active detection algorithm that adapts the classifier for its application in real-time fatigue detection. This dynamic adaptation of the fatigue score threshold turns users into their own controls over time by carrying information from previous estimates to generate the present score. It is one of the main differentiators of this work from prior state-of-the-art approaches to this problem, which generally use a cross-sectional design to evaluate fatigue at a given time point [38-40]. As an example, Ulinskas et al [38] assign fatigue levels to users’ data based on the time of the day. Morning, afternoon, and evening data generated by the same user are treated as independent samples in a multivariate classification framework that ignores the sequential relation between fatigue states over daily cycles. The classification results in the controlled experiment (ie, accuracy in the separation of rested vs fatigue samples in the sleep inertia data set) are worse than the ones presented in previous work completed using the same data set [27]. However, this approach reduces significantly the size of the input sample, 150 keystroke sequences (<1 minute at average speed) in comparison with the 15-minute long typing samples used in the study by Giancardo et al [27]. The value of this parameter is critical for the applicability of fatigue detection via keystroke monitoring in a real-world setting, as users are unlikely to generate continuous 15-minute long typing samples on their daily use of computer keyboards. When applied on an independent data set comprised by natural typing data collected in a real-world environment, the population-level results align with the results presented in previous studies on daily sleep and alertness cycles [41], which suggest high alertness during daytime, peaking a few hours after awakening, and higher sleepiness during nighttime.

In general, our results suggest that users are usually more awake and active during the mornings. Fatigue appears generally during the afternoon and increases as the day gets closer to regular sleep times. The daily averaged scores suggest a subtle fatigue peak after midday that could be associated with what has been referred in the literature as postlunch dip in performance [42]. This consensus with sleep and performance studies supports our hypothesis that keystroke dynamics can be used to quantify daily fatigue in computer users in an objective and unobtrusive manner. However, it is important to note that these daily cycle results have been analyzed at a population level and are not considering the variability in participants’ personal routines and schedules. Future studies pairing keystroke with other high-frequency fatigue–related data (eg, sleep and activity) could help us better assess the performance of the proposed method at user level.

As for its clinical application, fatigue is a common symptom that can precede or reflect the presence of a more serious mental or physical condition. The current standard to clinically assess fatigue relies on patient-reported outcomes through standardized questionnaires, such as the Fatigue Severity Scale [43]. To identify fatigue as a symptom, patients must first identify unusually excessive fatigue patterns and then alert their physician before it can be further investigated. This leaves fatigue as a commonly overlooked or unrecognized predictor of other emerging disorders [3,4]. Fatigue has also been reported as a frequent side effect of disease treatment [44] and long-term sequel of conditions such as COVID-19 [45].

The proposed methodology is designed to validate an approach for objective and passive fatigue monitoring. Leveraging the widespread use of PCs, this framework presents an opportunity to provide more visibility and accurate tracking of fatigue and its clinical implications. As it runs in the background of users’ computers, this approach could potentially be used to alert patients and health care professionals of early signs of abnormal fatigue to uncover progressive disease or the presence of underlying conditions. In the context of clinical trials or during disease management, this method could also be used to enable objective and real-world evaluation of the impact of newly developed or existing treatment regimens on a patient’s fatigue state.

As a major limitation of this work, the fatigue detection model performs better for some participants than others because of the intrauser variations when typing [33]. In users who show little variation between resting and fatigue states, the model does not effectively classify performance. An example of this is shown in Figure 3, where we can observe a clear separation between the rest keystroke sessions and the fatigue ones for the participants (Figure 3A), meanwhile the fatigue detection model struggles when trying to separate the keystroke sessions for the participants of the Figure 3B with poor results.

### Conclusions

This work presents a step toward the development of a real-world fatigue monitoring tool that operates passively by leveraging users’ natural interaction with their PCs. It is important to note that the data set used in these analyses is composed solely of healthy controls; future work should evaluate the performance of the proposed method in a cohort that includes participants with conditions affecting psychomotor health that may mask or be confounded by fatigue symptoms. Another limitation and potential line for future research is that this work has been tested using mechanical keyboard data, thus future applications of this specific methodology require users who type frequently on mechanical keyboard devices. Adapting this framework to include touchscreen devices would expand the population that could benefit from this method. Given that typing kinematics vary significantly between mechanical and touchscreen devices, this adaptation would require additional studies. The limited dimension of the sleep inertia database is another aspect to take into account in future studies. Although the use of domain adaptation techniques reduces the need for larger supervised data sets, increasing the size of the controlled cohort would allow for optimization of the target task layer and independent validation of the fatigue detection classifier. Finally, although the crowdsource results are similar to previously published studies on daily alertness, full validation would require a labeled real-world data set to test the generalizability of the proposed framework for its application in the real work setting. Additional validation in specific use case scenarios would pave the way for use of this method as an objective, high-resolution, and quasicontinuous way to monitor users’ fatigue with minimal burden on their daily routine.

## Abbreviations

ADD: average detection delay
AFD: active fatigue detection
AUC: area under the curve
DML: distance metric learning
DNN: deep neuronal network
EER: equal error rate
k-NN: k-nearest neighbor
LOO: leave-one-out
nQCS: neuroQWERTY Crowdsource
nQSI: neuroQWERTY Sleep Inertia
PFD: probability of false detection
PND: probability of nondetection
RF: random forest
SVM: support vector machine
